# Functional Studies of *MLC1* Mutations in Chinese Patients with Megalencephalic Leukoencephalopathy with Subcortical Cysts

**DOI:** 10.1371/journal.pone.0033087

**Published:** 2012-03-05

**Authors:** Han Xie, Jingmin Wang, Ajit Singh Dhaunchak, Jing Shang, Liping Kou, Mangmang Guo, Ye Wu, Qiang Gu, David Colman, Xiru Wu, Yuwu Jiang

**Affiliations:** 1 Department of Pediatrics, Peking University First Hospital, Beijing, People's Republic of China; 2 Montreal Neurological Institute, Montreal, Quebec, Canada; 3 Shanxi Medical University, Taiyuan, Shanxi, People's Republic of China; Brigham and Women's Hospital, Harvard Medical School, United States of America

## Abstract

Megalencephalic leukoencephalopathy with subcortical cysts (MLC, MIM# 604004) is an autosomal recessive inherited disease mostly resulting from *MLC1* mutations. In this study, we finished the functional analysis of *MLC1* mutations identified recently in Chinese patients, including five newly described missense mutations (R22Q, A32V, G73E, A275T, Y278H), one known nonsense mutation (Y198X), and two known missense mutations (S69L, T118M). We found MLC1*^wt^* was localized to the cell periphery, whereas mutant R22Q, A32V, G73E, S69L and T118M were trapped in the lumen of endoplasmic reticulum (ER) when we transfected the wild-type and mutant MLC1 in U373MG cells. Compared to wild type, the mutant G73E, T118M, Y198X and A275T transcript decreased and all mutants except R22Q had lower protein expression in transfected U373MG cells. Therefore, we propose that all these eight *MLC1* mutations had functional effect either on their protein/mRNA expression, or on their intracellular protein localization, or both.

## Introduction

Megalencephalic leukoencephalopathy with subcortical cysts (MLC, OMIM 604004) is a rare congenital vacuolating leukodystrophy characterized by early-onset macrocephaly at birth or during the first year of life. Ataxia, seizures, and usually later onset mild mental deterioration are other common clinical features [1]. Representative MRI shows diffusely abnormal white matter with subcortical cysts in the tips of the temporal lobes and in frontoparietal subcortical areas [2].

MLC is a genetically heterogeneous condition resulting from gene defects either in *MLC1 or HEPACAM*. *MLC1* (GenBank NM_015166) is mapped to chromosome 22q13.3, containing 12 exons with a start codon in exon 2 and an 2.2 kb untranslated 3-prime end [3]. MLC1 is highly expressed in cerebellum, olfactory tract, brainstem and thalamus, but has weaker expression in cerebral cortex, striatum, and hippocampus [4]. MLC1 mainly presents in astrocytes, but not in oligodendrocytes. Specifically, MLC1 localizes in astrocyte-astrocyte membrane contact regions [5]. At the subcellular level, human MLC1 localizing in the plasma membrane forms eightmers (oligomers) and is predicted to span the plasma membrane eight times [6]. MLC1 is homologous with carrier proteins and is confined to the plasma membrane, which indicates that it may regulate substance translocation across the cell membrane [3], [7]. Besides, it has not yet been determined whether MLC1 is localized in membrane contact regions between endothelial cells and glial cells or between different kinds of glial cells [8]–[10].

Although the exact function of MLC1 remains unclear, the analysis of its Amino acid sequence reveals a slight similarity with potassium channel Kv1.1, ABC-2 type transporters and sodium-galactoside symporters [3], [6]. MLC1 has been recently shown to regulate the chloride current and cell volume in astrocytes consistent with its structural homologies to an ion channel [11], [12].

In addition, many *MLC1* mutations have been identified in the past years [3], [13]–[19]. Until now, there are around 70 MLC-related mutations of *MLC1* have been reported in patients of various ethnic backgrounds (human gene mutation database, HGMD). Despite this, families without identifiable mutation at the *MLC1* locus had been found [3], [20], and the existence of at least one other locus had been suggested before [21], [22]. Recently, López-Hernández T et al. found mutations in GlialCAM encoded by *HEPACAM* in some MLC patients without *MLC1* mutations. Further experiments demonstrated that GlialCAM was essential for accurate localization of MLC1 [23]. Thus, *HEPACAM* is the second gene associated to MLC.

In our previous study, we identified 10 *MLC1* mutations in 13 Chinese patients, including five newly described missense mutations (c.65G>A, p.R22Q; c.95C>T, p.A32V; c.218G>A, p.G73E; c.823G>A, p.A275T; c.832T>C, p.Y278H), one newly described splicing mutation (c.772-1G>C in IVS9-1), one newly described small deletion (c.907_930del, p.V303_L310del), one known nonsense mutation (c.593delCTCA, p.Y198X) and two known missense mutations (c.206C>T, p.S69L; c.353C>T, p.T118M) [24]. In this study, we carried out the functional analysis of eight MLC1 mutants (not involving c.772-1G>C in IVS9-1; c.907_930del, p.V303_L310del) to investigate the pathogenesis of MLC. With the exception of T118M, seven of the eight mutations had not been studied functionally before. All these eight mutations were analyzed by mRNA, protein expression and intracellular protein localization in this study. We used the mutant T118M as a positive control in our study according to the previous report which demonstrated that the mutant T118M declined in MLC1 plasma membrane protein [6].

## Results

### Intracellular localization of wild-type and mutant MLC1

**Figure 1:**
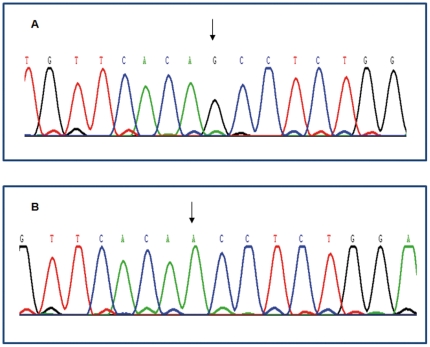
The sequencing results of MLC1*^wt^* and the mutant. (A) The sequencing result of MLC1*^wt^*. Arrow showed cDNA 823 base was G; (B) The sequencing result of c.823G>A. Arrow pointed G changed to A (Other data not shown).

Seven missense (c.65G>A, p.R22Q; c.95C>T, p.A32V; c.218G>A, p.G73E; c.823G>A, p.A275T; c.832T>C, p.Y278H; c.206C>T, p.S69L; c.353C>T, p.T118M) and one nonsense mutation (c.594delCTCA, p.Y198X) were generated by site-directed mutagenesis. Fluorogram of MLC1^wt^ and mutant allele (A275T) resulting from G to A transition at position 823 in MLC1 cDNA were shown in Figure 1.

**Figure 2:**
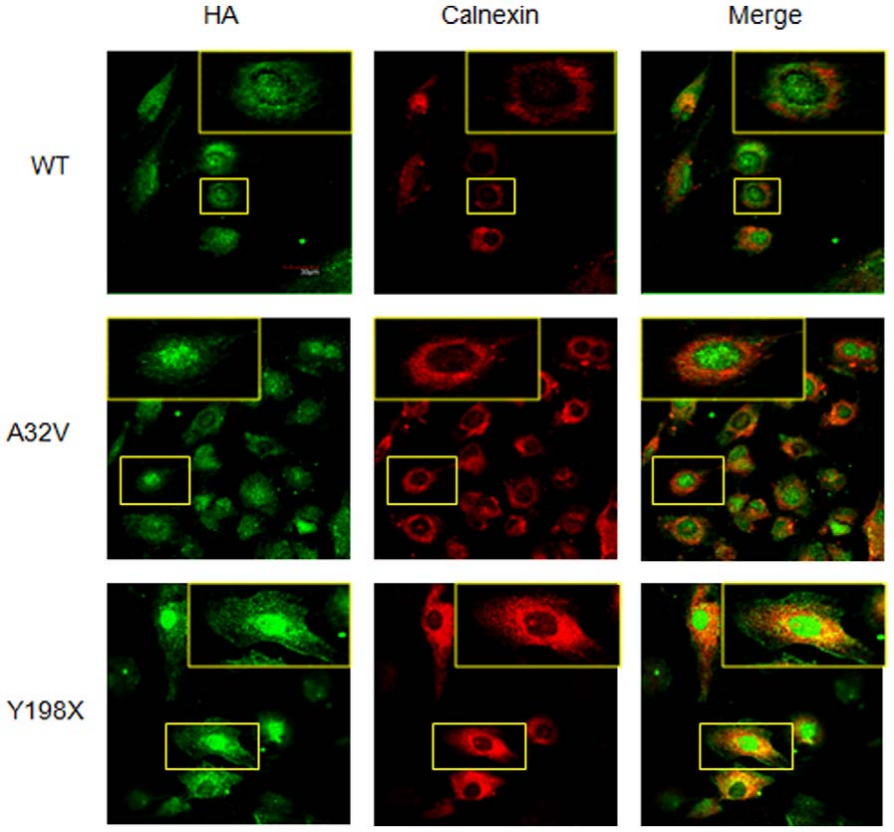
Some MLC1 mutants were retained intracellularly and co-localized with an ER marker. Confocal images of transfected U373MG cells expressing various MLC1 mutants immunostained with anti-HA antibody (green) and calnexin (red) were shown (magnified in Inset). Mutant MLC1 staining mostly overlapped with calnexin for A32V. Y198X showed minimal co-staining with calnexin and was expressed like MLC1*^wt^*. The bar was 30 µm (Other data not shown).

**Figure 3:**
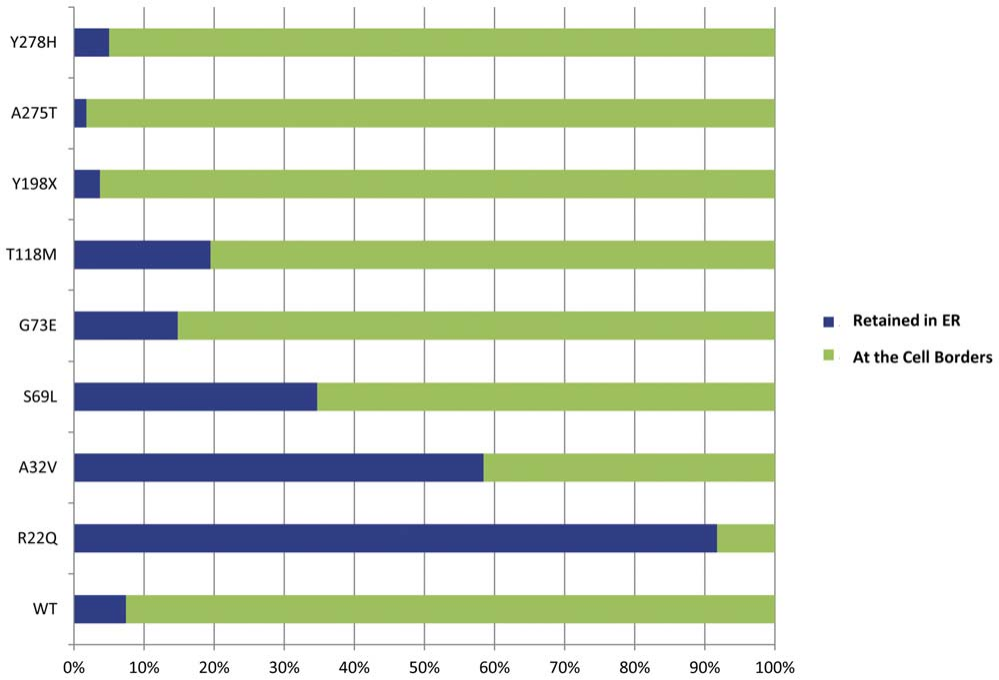
Transfected cells were scored for MLC1 subcellular protein distribution at the cell periphery or peri-nuclearly. MLC1*^wt^* was mainly at the cell border (92.60% cells), whereas *MLC1* mutants R22Q, A32V, S69L, G73E and T118M were mainly ER retained, standing at 91.74%, 58.43%, 34.71%, 14.81% and 19.48%, respectively. Surprisingly, A275T, Y278H and Y198X were found at the cell borders (n = 3 experiments).

In transfected cells, MLC1*^wt^* distributed to cell periphery whereas mutant MLC1 was retained in the perinuclear sub-compartment. Upon co-staining with ER marker, calnexin, we found co-localization of calnexin with mutant MLC1 in transfected U373MG cells. As shown in Figure 2 and Figure 3, MLC1 R22Q and A32V mutants staining mostly overlapped with calnexin. Other mutants, S69L, G73E and T118M only showed mild retention and were not entirely confined to the perinuclear space. However, MLC1 Y198X, A275T and Y278H mutants showed minimal co-staining with calnexin and had expression pattern similar to that of MLC1*^wt^*.

### Protein expression of MLC1

**Figure 4:**
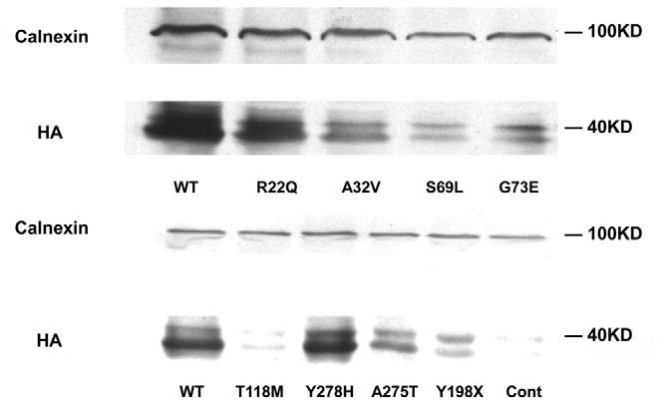
Protein expressions of MLC1*^wt^* and the mutants. Calnexin was an internal control and MLC1 proteins were tagged by HA. Cont was the control without transfection.

**Figure 5:**
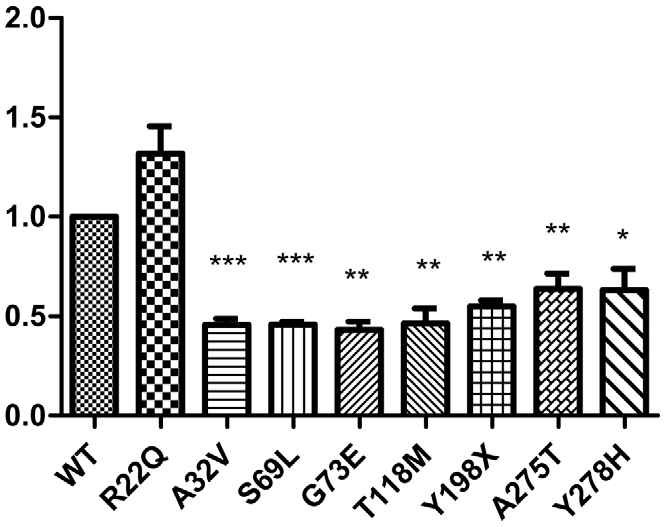
Statistics for protein expressions. We defined WT as 1. Except for R22Q, all other mutations resulted in significant downregulation in mutant MLC1 protein levels (n = 4). The statistics of these data was compiled by T test in Prism 5 (*** *P<0.001*, ** *P<0.01*, * *P<0.05*, compared with WT).

A32V, G73E, S69L, T118M, Y198X, A275T and Y278H mutants showed a decrease in MLC1 protein expression. Compared to the wild-type MLC1 (defined as 1), A32V reduced to 0.456, G73E to 0.432, S69L to 0.458, T118M to 0.488, Y198X to 0.545, A275T to 0.637 and Y278H to 0.632, however, R22Q had no significant change in statistic analysis (*P>0.05*). These were shown in figure 4 and figure 5.

### mRNA expression of MLC1

**Figure 6:**
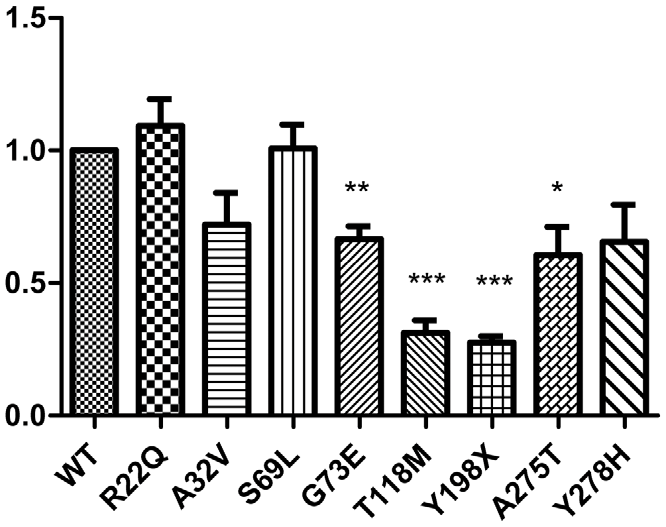
mRNA expressions of MLC1*^wt^* and the mutants. SYBR-Green quantitative real-time PCR analysis revealed that, when compared with MLC1^wt^, MLC1 mutants (G73E, T118M, Y198X, and A275T) were expressed at significantly lower levels. No significant changes were observed in R22Q, A32V, S69L and Y278H (n = 3).The statistics of these data was compiled by T test in Prism 5 (*** *P<0.001*, ** *P<0.01*, * *P<0.05*, compared with WT).

By defining WT as 1, we observed that transcripts of MLC1 mutants R22Q (1.093) and S69L (1.007) were expressed to same levels as that of WT. Moreover, mutants A32V and Y278H had no significant changes (*P>0.05*). In addition, G73E decreased significantly to 0.665, T118M to 0.311, Y198X to 0.276, and A275T to 0.606 (figure 6).

## Discussion

*MLC1* is the first identified and main causative gene known to be associated with MLC. Sequence analysis identified mutations in approximately 70% of clinically diagnosed MLC individuals. Since the identification of this causative gene in 2001, no studies have revealed the functional change of MLC1 variants found in non-Caucasian patients in terms of both mRNA and protein levels. Through a combination of western blot, real-time PCR and confocal imaging, we demonstrated that mRNA and/or protein expression of these *MLC1* mutants decreased, and the intracellular distribution of mutant MLC1 changed significantly. It seems that these changes contribute to the phenotype in MLC patients.

### MLC1 mutants accumulated in the ER

*MLC1* has been proven to be expressed in brain, and alternative splicing products have not been detected [3]. *MLC1* encodes a membrane protein that may transport a specific substrate and MLC1 is predicted to be an oligomeric protein involving 8 transmembrane segments. The forecasted transmembrane domain 4 and 8 include a fraction of some leucine residues, which indicates that leucine-leucine interaction plays a pivotal role in the oligomerization, like other membrane proteins [6].

In the brain, MLC1 protein is predominantly expressed in astrocyte end-feet, connecting with lipid rafts and the dystrophin glycoprotein complex (DGC). DGC is a multiprotein complex modulating brain development as well as ion and fluid homeostasis in astrocytes and neurons [25]. Intracellular domains of MLC1 are associated with the DGC proteins syntrophin, dystrobrevin, Kir4.1 and caveolin-1, the structural protein of caveolae [26]. The caveolar-lipid rafts that MLC1 localizes in are crucial for protein trafficking [27], [28]. Moreover, MLC1 is expressed in intracellular vesicles and endoplasmic reticulum, and is concerned with the endocytosis regulated by caveolae/raft. Inhibition of endocytosis, cholesterol diminution, and MLC1 phosphorylation improve the membrane-associated MLC1 expression [26]. However, pathological mutations prevent MLC1 membrane expression [6]. At this study, we detected that these eight MLC1 mutants except for Y198X, A275T and Y278H, unlike MLC1*^wt^*, were retained to different degrees in the ER. We conclude that most of the MLC1 mutant proteins are trapped in the ER, thereby inhibiting them to move to the cell membrane and carry out their functions

### Reduced mRNA and protein expression of the *MLC1* mutants

MLC1 protein is located in astrocytic processes related to blood and cerebrospinal fluid-brain barriers. The performance in cultured rat astrocytes illustrated that MLC1 was expressed in cell-cell contacts, which contains important proteins located in tight and adherent junctions. A MLC cell model was established by knockdown of MLC1 in primary astrocytes. Reduced MLC1 expression on this model led to the appearance of intracellular vacuoles. But the co-expression of human wild MLC1 can rescue the vacuolation. Moreover, a human brain biopsy of the MLC patient demonstrated that vacuoles were also found in astrocytic processes [29]. Thus, decrease in expression of MLC1 can clearly affect the structure of astrocytes.

Two previous studies with ten different MLC1 mutations in total [6], [30] showed that mutations mostly led to the reduction of protein expression. Another research showed that mutation C326R reduced MLC1 mRNA expression [20]. Further, decline of MLC1 protein and mRNA expression had also been found in monocytes from MLC patients [31]. In our study, most of mutations made MLC1 protein and/or mRNA expression decreased. That means the function of MLC1 should be affected by the reduction of *MLC1* expression caused by mutations. Interestingly, compared with MLC1*^wt^*, mutant A32V, S69L and Y278H expressed normal amounts of mRNA, but had lower protein expression. Further studies are warrant to investigate this at the translational level.

### Reductions of functional MLC1 proteins caused by mutants

MLC1 is mostly expressed in astrocyte–astrocyte junctions of blood– and CSF− brain barriers, Bergmann glia and main axonal tracts [5], [8], [9]. The GlialCAM is regarded as an MLC1 beta-subunit and affects MLC1 directly in astrocyte–astrocyte junctions. *HEPACAM* mutations also interact with the trafficking of MLC1, which causes the same disease MLC [32]. Moreover, most of MLC1 mutations led to reduced membrane expression of the MLC1 protein [31]. Thus adequate expression and proper positioning of MLC1 proteins is required to carry out their function effectively. For instance, although R22Q had enough expression, only 8.26% of the proteins ultimately were transported to the membrane. Consequently, R22Q showed an overall decrease in functional proteins. Moreover, compared with WT, the three mutants Y198X, A275T and Y278H seemed to have a slightly higher rate of proteins at the membrane according to the figure 3, but protein expressions in these three mutants were apparently reduced. Other mutants had not only reduction of expression but also abnormal localization. In brief, they are all devoid of enough functional proteins in the cell membrane.

In summary, we have finished the functional studies of eight *MLC1* mutants detected in Chinese patients, including Intracellular localization of wild-type and mutant MLC1, and translational and transcriptional change of mutants in transfected primary rodent astrocytes and brain epithelial derived cell line, U373MG. Mutant R22Q, A32V, G73E, S69L and T118M, unlike MLC1^wt^, were retained in ER. Moreover, we have identified significant decline in mutant transcripts and protein levels. Therefore, we conclude that these eight MLC1 mutations in Chinese patients decrease MLC1 protein and/or mRNA expression and disrupt intracellular protein localization, and these may be the molecular mechanisms related to their MLC phenotype.

## Materials and Methods

### Chemicals

Tris HCL1M (pH 6.8) cat. MC030.5 and Tris HCL 1.5 M (pH 8.8) cat. MC030.4 were from M&C Gene Technology. α-MEM cat. C0008 and D-PBS cat. SH0021 were from Beijing Four-Ring Sunny Bioscience CO.LTD. The U373MG cell line was obtained from Shanghai Fumen Company in China. pfu DNA Polymerase cat. M7741 was from Promega Corporation (USA). HA (Rabbit polyclonal antibody to HA tag) and HRP conjugated goat anti rabbit/mouse IgGs were from Gene Tex. Calnexin (TO-5) was from SANTA CRUZ BIOTECHNOLOGY.INC.

### Mutant human MLC1 constructs

Epitope tagged (HA) pcDNA3.1-MLC1 was kindly provided by M. S.van der Knaap (VU University Medical Center, Amsterdam, The Netherlands). Seven missense (c.65G>A, p.R22Q; c.95C>T, p.A32V; c.218G>A, p.G73E; c.823G>A, p.A275T; c.832T >C, p.Y278H; c.206C>T, p.S69L; c.353C>T, p.T118M) and one nonsense mutations (c.594delCTCA, p.Y198X) were generated by PCR site-directed mutagenesis. To generate the mutants, the oligonucleotide primers were used and shown in Table 1. All site-directed mutants were generated by circular amplification of this plasmid with PFU Turbo DNA polymerase (Stratagene, La Jolla, CA), followed by DpnI digestion (New England Biolabs, Ipswich, MA) and transformation into *Escherichia coli*. Individual clones were analyzed by DNA sequence analysis.

**Table 1:** Oligonucleotide primers used to generate *MLC1* mutants.

*MLC1* mutants	Oligonucleotide Primers
G73E	5′-cgctgtacctggagaacgtgttccc-3′
	5′-gggaacacgttctccaggtacagcg-3′
A275T	5′-cgctgctgttcacaacctctggatatctg-3′
	5′-cagatatccagaggttgtgaacagcagcg-3′
Y198X	5′-gggtcctgaaatcttagtcgtcgaggtaatcg-3′
	5′-cgattacctcgacgactaagatttcaggaccc-3′
R22Q	5′-tggagcggggccagcaagaccccgc-3′
	5′-gcggggtcttgctggccccgctcca-3′
A32V	5′-atgccccagacgtgaagccgagcga-3′
	5′-tcgctcggcttcacgtctggggcat-3′
Y278H	5′-cctctggatatccgtcattcagcat-3′
	5′-atgctgaatgacggatatccagagg-3′
S69L	5′-cctcggggtttttgctgtacctggg-3′
	5′-cccaggtacagcaaaaaccccgagg-3′
T118M	5′-tgtttgtttccatgtttgctgtgac-3′
	5′-gtcacagcaaacatggaaacaaaca-3′

### Cell culture

U373MG cells were cultured in α-MEM containing 10%FBS (fetal bovine serum) and antibiotics: streptomycin (25 µg/ml) and penicillin (25 U/ml). Cells were maintained in a humidified incubator at 37°C in 5% CO_2_ atmosphere. The medium was exchanged every 48–72 h. Cells were passed by 0.25% trypsin every 3 days.

### Transfections

For transfection, 10 µl of Lipofectamine 2000 (Invitrogen, Carlsbad, CA) and 4 µg of plasmid DNA were incubated separately in Opti-mem (GIBCO Invitrogen, Carlsbad, CA) for 5 min at room temperature, then combined for another 20 min. U373MG cells (approximately 80–90% confluent) were washed with PBS, incubated with the combined Lipofectamine 2000/DNA solution in Opti-mem for 4–5 h at 37°C, then fed with α-MEM supplement with 10% FBS. After 24 h, transfected cells were either processed for western-blot, confocal microscopy, or real-time PCR.

### Western-blot

Transfected U373MG were washed thrice with ice-cold PBS and lysed in ice-cold lysis solution (2× lysis solution consisted of Triton X-100 0.4 ml, NaCl 0.18 g, 0.5 M EDTA 0.4 ml, 1 M Tris HCl (pH = 8.8) 2 ml, dH_2_O 10 ml) at 4°C for 20 min. After scraping with ell scraper, the lysates were collected and centrifuged at 12000 g for 30 min at 4°C. Finally liquid supernatant was stored at −80°C. Proteins were separated on 10% SDS polyacrylamide gels and electrotransferred (BioRad) to PVDF membranes. The membranes were blocked with 5% milk power in PBS, and probed with the indicated primary antibody by overnight incubation at 4°C. Four washes in 0.05% PBS-T (0.5 ul Tween-20 in 1 ml PBS) of 10 min each at room temperature were performed before incubation with secondary antibody. After four washes in 0.05% PBS-T of 10 min at room temperature, antigen-antibody complexes were visualized by enhanced chemiluminescence and exposed on X-ray films.

### Real-time PCR

Total RNA isolated by TRIzol Reagent (Invitrogen, Carlsbad, CA, USA) was used to eliminate the genome DNA contamination. Total RNA (1 ug) was reverse transcribed using reverse transcription kit (Promega, Madison, WI, USA). Quantitative real-time PCR was performed on ABI 7300 PCR Instrument (ABI, Foster City, CA, USA) with SYBR Green-real-time PCR master mix kit. GAPDH was used as the endogenous control. Forward (F) and reverse (R) primer sequences were shown in Table 2. PCR was performed for 5 min at 95°C, then 30 sec at 95°C and 30 sec at 55°C for 40 cycles.

**Table 2:** Primers used in real-time PCR.

Primer Names	Primer sequences
MLC1cDNA(a)-1 F	TCAGATATTGTTTGTTTCCACG
MLC1cDNA(a)-1 R	CAGGATGAGGTTGAAGTTGATG
MLC1cDNA(a)-2 F	GGAGGAACGCCAATGTG
MLC1cDNA(a)-2 R	GACCCGAGCAGGAAATG
MLC1cDNA(b)-1 F	CAAGGAGAAAGCCTGGAGAG
MLC1cDNA(b)-1 R	CAGTAGCTCAGGGCGATTAG
MLC1cDNA(b)-2 F	ATGTGGCAGCAGAGTGTCC
MLC1cDNA(b)-2 R	GCTGGCGGGTAATCCTT
GAPDH F	GAAGGTCGGAGTCAACGG
GAPDH R	CTCGCTCCTGGAAGATGG

GAPDH was used as the endogenous control. F: Forward Primer; R: reverse primer.

### Confocal microscopy

Confocal microscopy was carried out after transfection had been accomplished for 24 h. All steps were performed at room temperature, unless stated otherwise. Cells grown on confluent plates were washed three times briefly with PBS, fixed for 15 min in 4% (wt/vol) paraformaldehyde/PBS. After washed 3 times for 10 min each in PBS, cells were blocked in 2% (wt/vol) BSA for at least 60 min. A mouse anti-calnexin monoclonal antibody and a Rabbit anti-HA Polyclonal antibody respectively diluted 1∶2000 and 1∶5000 in 0.05% TBS were applied overnight at 4°C. After three washes in PBS (10 min each), fluorochrome-conjugated secondary antibodies were applied for at least 60 min. After three washes in PBS (10 min each), coverslips were mounted with Aqua-Poly/Mount (Polysciences Warrington, PA) on glass slides. Fluorescent images were captured on an Olympus confocal microscope (FV-1000 spectral-type) with a 63× oil Plan Apochromat objective.
